# On the External Use of Iodine in Croup

**Published:** 1849-06

**Authors:** 


*On the external use of Iodine in Croup.*—Dr. Willige speaks of having had remarkable success in the treatment of urgent cases of croup by the external application of iodine to the larynx and trachea. He recommends that tincture of iodine should be smeared with a feather over the front part of the neck, corresponding to the larynx and trachea and their immediate neighborhood; and that this should be repeated several times, with intervals of about four hours, until redness and irritation of the skin is induced. In most cases this is followed by subsidence of the distress of breathing, of the spasms of the glottis, and of the other bad symptoms. He mentions the particulars of three cases in which, by this means, he succeeded in averting impending death.—*Schmidt's Jahrbücher.—Southern Med. and Surg. Jour.*

